# Recto-Vaginal Fistula Cured without Operation

**Published:** 1880-06-20

**Authors:** Ira Brown

**Affiliations:** Wells River, Vt.


### RECTO-VAGINAL FISTULA CURED WITHOUT OPERATION.

BY IRA BROWN, M.D., WELLS RIVER, VT.

September 21, 1878, I was called to see J. L., a maiden lady about forty-eight years of age. She had been an invalid most of the time for twenty-five years, and now complained of frequent micturition, the water having a bad odor and containing a gritty dark sediment.

On making a digital examination, I found a recto-vaginal fistula twelve lines in length, with rounded and thickened edges, showing that it had existed for some time.

Not having the means at hand nor any one to assist in an operation, after introducing the speculum I made a thorough application to the edges of the fistula of a strong solution of carbolic acid, four parts of the acid to one part alcohol. This done the patient was directed to remain in bed, and to use no means to move the bowels until I saw her again.

September 25th, four days after the first visit, she reported herself better, her water having lost its bad odor and containing no sediment.

She was again seen September 29th, eight days from first visit, when an examination showed the fistula nicely closed in its whole extent.

October 9th, eighteen days from the first visit, a careful examination failed to show any trace of the fistula.

In July following, I learn that she had remained well. I would here venture the opinion that carbolic acid may be used in most cases instead of the knife in preparing surfaces to be united with sutures.—*Boston Med. and Surg. Journal.*

---

A Boston paper wickedly says : When a physician regards a case as hopeless he advises the patient to travel, and thus gets rid of having the victim die under his care.

Medical man : And then, with regard to the swelling at the back of your head, I don't apprehend anything serious, but you must keep your eye on it!

